# Ventriculoperitoneal Shunt Malfunction: The Importance of Topogram Image Observation

**DOI:** 10.1002/ccr3.72570

**Published:** 2026-04-23

**Authors:** Yantao Du, Xiongwei Ding, Wenquan Wang, Yanhui Du

**Affiliations:** ^1^ Department of Radiology Dezhou Seventh People's Hospital Dezhou China; ^2^ Department of Radiology Jinan High ‐ Tech East District Hospital Jinan China; ^3^ Department of General Surgery Dezhou Seventh People's Hospital Dezhou China; ^4^ Department of Pediatrics Shandong University Affiliated Shandong Provincial Third Hospital Jinan China

**Keywords:** CT topogram, hydrocephalus, sunset eye sign, ventriculoperitoneal shunt disconnection

## Abstract

Disconnection of a ventriculoperitoneal shunt is a serious complication. The “sunset eye” sign is an important indicator of hydrocephalus. Observation of a CT topogram is an important diagnostic measure and should not be overlooked, and good care of the ventriculoperitoneal shunt should also be taken.

A 16‐year‐old boy was diagnosed with grade I astrocytoma after sudden syncope and underwent tumor resection. He subsequently developed hydrocephalus and intracranial infection (no pathogen testing was performed), underwent ventriculoperitoneal (VP) shunt placement approximately 1 year prior. Three days ago, during class, he suddenly experienced dizziness and headache, which progressively worsened and were accompanied by nausea; multiple self‐adjustments of the pressure valve did not relieve his symptoms. On physical examination, downward deviation of both eyeballs with exposed upper sclera was noted, resembling the “setting sun” appearance, also known as the “sunset eye” sign. Cranial CT scanning revealed significant dilation of the ventricular system, indicating the presence of hydrocephalus, which was further confirmed by comparison with the patient's prior routine follow‐up CT images. CT topogram demonstrated an abrupt interruption of the occipital VP catheter with no visible drainage tube, confirming VP shunt disconnection (Figure 1A, arrow). Abdominal X‐ray showed the VP catheter coiled in the pelvis, completely detached from its connection site (Figure 1B, arrow). The patient subsequently underwent VP shunt revision surgery; the postoperative CT topogram confirmed reconnection of the VP catheter (Figure 1C, arrow), with concurrent resolution of his symptoms. At the 2 week follow‐up, he had returned to normal life. The boy remains in good health to date, 1 year postoperatively.

**FIGURE 1:**
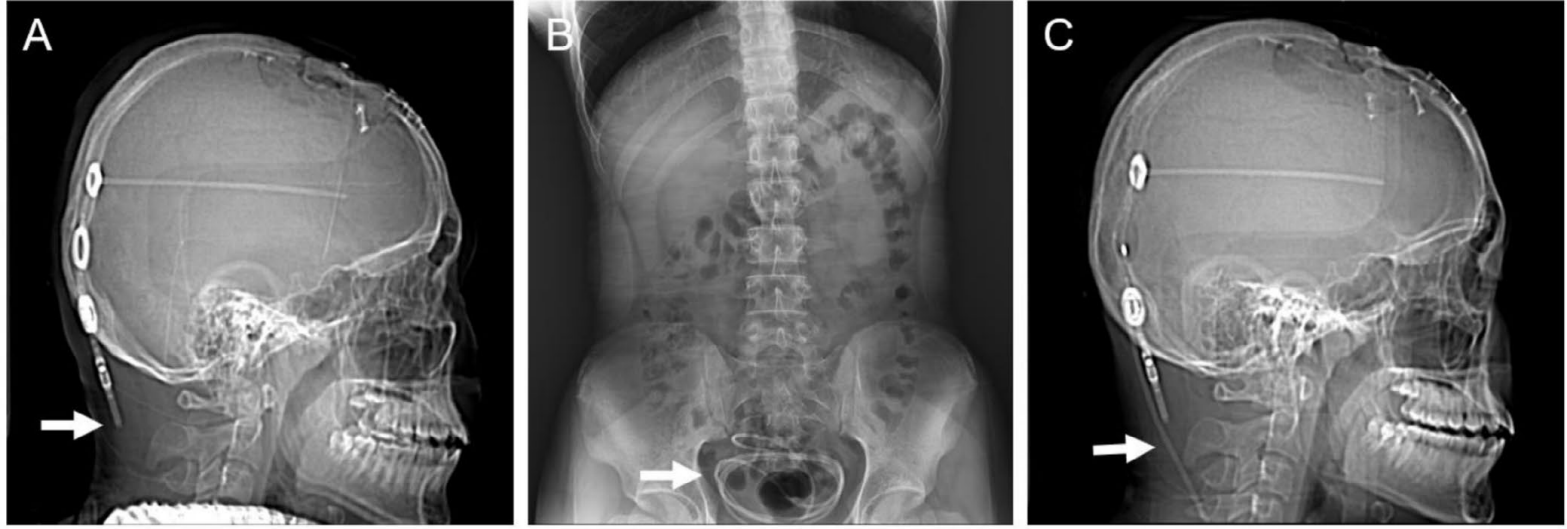
(A) CT topogram demonstrated an abrupt interruption of the occipital VP catheter with no visible drainage tube, confirming VP shunt disconnection (white arrow). (B) Abdominal X‐ray showed the VP catheter coiled in the pelvis, completely detached from its connection site (white arrow). (C) CT topogram demonstrated reconnection of the VP catheter after revision surgery (white arrow).

The “sunset eye” sign is a classic manifestation of hydrocephalus [1], often associated with symptoms such as drowsiness or lethargy. VP shunt placement remains a common treatment for hydrocephalus; however, complications such as shunt obstruction, infection, and overdrainage are relatively frequent; the disconnection rate of VP shunts in children is approximately 3.6%. While shunt devices are generally durable, growth and development, surgical techniques, drainage tube aging, intraperitoneal placement sites, and strenuous exercise are common causes of shunt fracture. Malfunction after magnetic resonance imaging has also been reported [2]. This boy presented with acute onset and rapid symptom deterioration, following a recent episode of strenuous exercise (badminton). Over time, the catheter may degrade and calcify, leading to fractures typically occurring years after implantation; the mean duration until diagnosis of discontinuity was 66.3 ± 24.1 months. Notably, such strenuous exercise is the predominant factor leading to early VP shunt fracture—consistent with the present case. Identifying the underlying cause is critical for effective treatment; blindly adjusting the pressure valve will not alleviate symptoms and may result in continued intracranial pressure elevation, worsening the patient's condition; against this backdrop, telemetric intracranial pressure monitoring is particularly important, enabling earlier detection of elevated intracranial pressure [3]. Radiologists typically prioritize axial images on cranial CT to assess intracranial abnormalities; axial images can confirm hydrocephalus but not its root cause, whereas the frequently neglected CT topogram—with its panoramic visualization of the entire shunt pathway—serves as a key diagnostic tool, and failure to utilize it may result in diagnostic delays, underscoring the importance of etiological identification for targeted treatment. Meticulous maintenance and follow‐up of the VP shunt are essential.

## Author Contributions

**Yantao Du:** project administration, resources, writing – original draft, writing – review and editing. **Xiongwei Ding:** resources, visualization. **Wenquan Wang:** writing – review and editing. **Yanhui Du:** conceptualization, methodology, validation.

## Funding

The authors have nothing to report.

## Ethics Statement

All procedures performed in this study were in accordance with the ethics standards of the institutional and/or national research committee and with the 1964 Helsinki Declaration and its later amendments or comparable ethics standards.

## Consent

Written informed consent was obtained from the patient to publish this report in accordance with the journal's patient consent policy.

## Conflicts of Interest

The authors declare no conflicts of interest.

## Data Availability

The data that support the findings of this study are available from the corresponding author upon reasonable request.
